# Time Resolved Raman Scattering of Molecules: A Quantum Mechanics Approach with Stochastic Schroedinger Equation

**DOI:** 10.1021/acs.jpca.2c05245

**Published:** 2022-10-24

**Authors:** Giulia Dall’Osto, Stefano Corni

**Affiliations:** † Department of Chemical Sciences, University of Padova, via Marzolo 1, Padova, 35131, Italy; ‡ CNR Institute of Nanoscience, via Campi 213/A, Modena, 41125, Italy

## Abstract

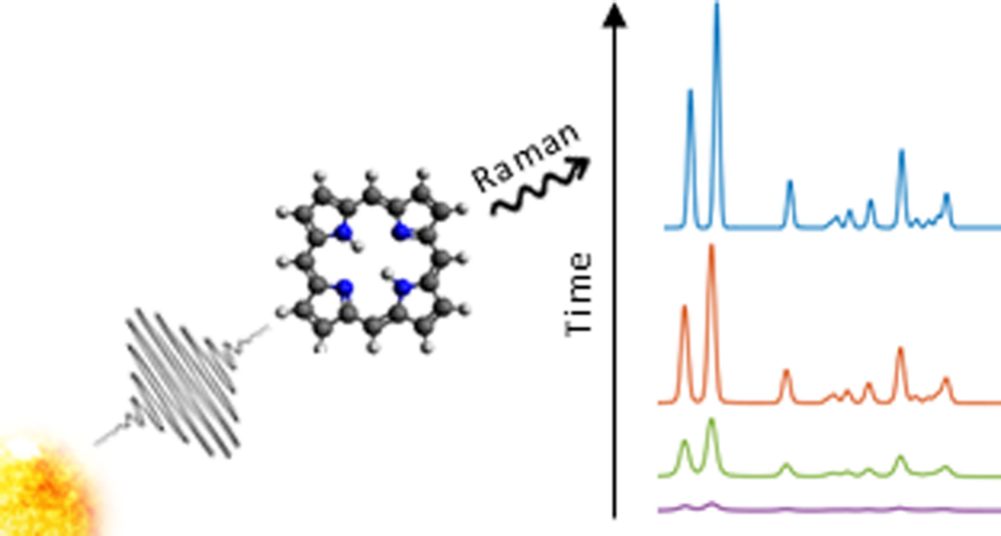

Raman scattering is a very powerful tool employed to characterize molecular systems. Here we propose a novel theoretical strategy to calculate the Raman cross-section in time domain, by computing the cumulative Raman signal emitted during the molecular evolution in time. Our model is based on a numerical propagation of the vibronic wave function under the effect of a light pulse of arbitrary shape. This approach can therefore tackle a variety of experimental setups. Both resonance and nonresonance Raman scattering can be retrieved, and also the time-dependent fluorescence emission is computed. The model has been applied to porphyrin considering both resonance and nonresonance conditions and varying the incident field duration. Moreover the effect of the vibrational relaxation, which should be taken into account when its time scale is similar to that of the Raman emission, has been included through the stochastic Schroedinger equation approach.

## 1 Introduction

One of the techniques mainly employed to investigate the vibronic structure of molecules is Raman spectroscopy,^1−5^ which has become a very powerful tool useful for the characterization of molecular systems.^6^ Due to the very short time scale of the process, the Raman signals are very sharp, and thanks also to the richness of information enclosed on the vibronic structure, they can constitute a fingerprint for molecules.^7,8^

What occurs in Raman scattering is a two-photon process^9,10^ that involves a first interaction with an incident radiation which makes the molecule explore a virtual state, namely a superposition of excited states, and the emission of a photon with energy that differs from the incident one by a molecular vibrational frequency.^11,12^ Considering an incident radiation in resonant conditions, the distinction is made between resonance Raman scattering and fluorescence emission that differ in time scale and spectral shape: Raman scattering is the early time emission (which generates spike lines in the spectrum) and the fluorescence is the late time emission (responsible for broad bands).^13^ In nonresonant conditions the Raman scattering and the fluorescence lay on a different range of the scattered energies and thus the distinction is unequivocal.

Raman scattering is distinguished from Rayleigh scattering, the elastic process, in which the scattered photon has the same energy of the incident one, or in other words the scattered photon brings the molecule back to the initial state. Raman process is related to the inelastic scattering of photons namely the scattered light has a different frequency with respect to the incident light. The bands generated by the Raman scattering are named Stokes when the scattered light has a frequency smaller than the incident light, otherwise they are called anti-Stokes. Differently from other spectroscopic techniques, the quantum mechanical description of the process is not trivial, and a great effort has been employed so far to give insight on the process and to theoretically reproduce the experimental results.

The theoretical methods employed to simulate Raman scattering start from the Kramers, Heisenberg, and Dirac (KHD)^14−16^ polarizability tensor and can be categorized in time-independent and time-dependent strategies. To the former belong the so-called sum-overstate methods based on an explicit expansion^17−20^ of the transition dipole moments ideally on all the molecular vibronic states.^21−24^ On the time-dependent side, the Lee and Heller^12,13^ theory gave a new picture of the Raman process as result of the propagation of a wavepacket on the excited-state potential energy surface. In this framework, the Raman intensity is determined by the half Fourier Transform of the overlap of initial and final states.^25^ Time-dependent strategies have been developed coupling Raman theory to DFT methods^26−29^ or employing Green’s function method to calculate the vibronic states overlap.^30^ On the other hand a real-time propagation can provide for the molecular response in the perturbation theory framework to retrieve the KHD polarizability.^31,32^ Other approaches developed in the time domain, based on the Fourier transform of the polarizability, have also increased the accuracy of results including the Herzberg–Teller vibronic coupling^33−35^ and the Duschinsky effect.^36,37^

In this work we propose a time-dependent procedure able to give a view of the process that goes on in Raman scattering, based on a semiclassical approach, that is, an approach which combines the classical treatment of the electric field with a quantum description of the system through its vibronic wave function.^38−42^ On the basis of the time-dependent Raman scattering theory proposed by Lee and Heller,^12^ a real-time propagation of the system wave function has been coupled with an integration in time^11^ that results in the computation of the time-dependent Raman cross-section through the calculation of the second order wave function. Compared with the original treatment of Lee and Heller,^12^ and subsequent TD approaches building on that, the main novelties are (i) we are using a numerically defined excitation pulse shape, without assuming a monochromatic continuous wave excitation; (ii) we are not only calculating the total Raman scattered intensity, but we provide the time-dependent cumulative signal; (iii) we include relaxation effects (and potentially also decoherence ones) via the Stochastic Schroedinger equation technique. The latter point allows treating Raman and fluorescence on the same footing, both resulting (on different time scales) from the same simulation.

Employing a generic electric field shape, which could be even defined by points, makes the simulation closer to pulsed experimental setups.

The present approach, which is not only time-dependent but also time-resolved, allows having information on the time scale of the process and also on the way Raman spectra are built in time in comparison with the time scale of the incident radiation.

Within this procedure, phenomena of decoherence and relaxation can be included in the dynamics through stochastic methodologies to consider the interaction with a surrounding environment in an effective way.^43−45^ In this work, the stochastic Schroedinger equation has been employed in order to investigate the role of the vibronic relaxation on the Raman process. Vibronic relaxation becomes relevant in the process since it occurs within a picosecond time scale that is also the time scale of the dynamics here reported.

The method here proposed can be applied without further approximations both in resonance and nonresonance conditions between the pulse frequency and the electronic transition. It is worth mentioning that with this strategy both the Raman signal and the fluorescence emission are computed since both contributions, in the present semiclassical formulation, come from the wave function at the second order with respect to the electric field even if the process is different. The scattered photon emitted in the Raman process causes the decay from a virtual state with energy determined by the pulse frequency to a vibronic state of the ground state, while the fluorescence emission needs, at first, to create a population on the excited state and so the emitted photon has energy close to the electronic transition. On this basis, the two phenomena can be distinguished when the two scattering signals are wide apart as in nonresonant conditions. Strategies to computationally tackle time-dependent frequency resolved spontaneous emission have been discussed in the past,^46−48^ as well as for time-dependent stimulated Raman;^49,50^ computational investigations of the time-development of spontaneous Raman spectra and its interplay with fluorescence received less attention.

This work is a prelude of the more challenging goal of computing the time-dependent spontaneous Raman scattering of molecules close to plasmonic nanoparticles, in order to simulate and predict surface-enhanced Raman scattering (SERS)^51−53^ experiments in a time-dependent perspective, combining the real-time dynamics of a molecule vibronic wave function with the propagation of the plasmonic nanostructures dielectric polarization.^44,54−56^

The paper is organized as follows: In section 2 we summarized the theoretical methods divided into the formulation of the time-dependent Raman scattering cross-section, the model for the system vibronic wave function employed, the numerical propagation procedure, and the computational details of the calculations performed; the results obtained with our model and the related discussion are presented in section 3 and final remarks are reported in section 4.

## 2 Methods

### 2.1 Time-Dependent Raman Scattering Formulation

In this section the main aspects of the theory we employed to describe Raman scattering are highlighted. The time-dependent picture of Raman scattering can be retrieved in the framework of perturbation theory through the two-photon formula. The first order correction to the system wave function allows a description of a one-photon process, such as absorption or emission. It is the result of the interaction between the time evolution of the unperturbed (initial) wave function and the electric field that induces a dynamics on an excited potential energy surface, which in atomic units reads as
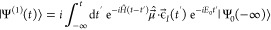
1|Ψ_0_(− *∞*)⟩ is the initial unperturbed wave function, considered here to be the ground state, with energy *E*_0_,  is the transition dipole moment operator,  is the incident electric field interacting with the system at time *t*′, and  is nuclear plus electronic Hamiltonian in the Born–Oppenheimer (BO) approximation. Note that we are also departing from the original Lee and Heller treatment (see also ref (11)) since there the propagation is done on electronic wave functions and  refers to the electronic Hamiltonian of the proper potential energy surface. Instead, here the vibronic Hamiltonian (i.e., the total Hamiltonian written on the basis of the vibronic wave functions in the BO approximation) enters in the propagation of the wave function. The BO approximation limits our approach to molecules where the vertical excitation region of the lowest excited state is sufficiently far from avoided crossings, where it would break down.^57−59^ To account for spontaneous emission from the virtual state that strictly speaking would require a quantum electrodynamics (QED) treatment, we shall use the same technique proposed by Lee and Heller.^12^ In practice, a second classical field  is considered, that is monochromatic at the frequency ω_*S*_, whose properties are then chosen to reproduce QED results. At the second order in the mixed  and  perturbation, neglecting the antiresonant term, one gets
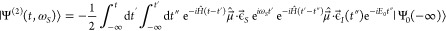
2which differs from the original Lee and Heller theory for the missing antiresonant term. In eq 2 the system wave function, after a dynamics guided by the Hamiltonian , interacts with  that brings the system at a low energy level, which then evolves under the vibronic Hamiltonian. The second electric field is not included explicitly in the dynamics since it is convenient to calculate its effect a posteriori. Neglecting the antiresonant term (that in which the field  interacts with the molecule before ) is an approximation that limits the validity of the present treatment to resonant and preresonant conditions, while it would be unsuitable (in terms of quantitative accuracy for the Raman intensities) for long wavelength excitations. Nothing fundamental prevents the inclusion of this term in our approach, but to be calculated for a generic  it would require several numerical propagations, starting from all the possible excited vibronic states, representing an increase of the computational burden not needed in the present work.

Equation 2 can be expressed as a function of the first order wave function as
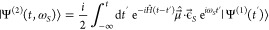
3where the dynamics of |Ψ^(1)^(*t*′)⟩, expressed by eq 1, can be obtained numerically through a real-time propagation of the initial wave function interacting with any shape of the incident electric field.

Practically, the wave function is expressed as a linear combination of vibronic eigenstates |*J*⟩
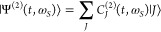
4weighted by the time-dependent coefficients at the second perturbative order. Multiplying left and right of eq 4 by the eigenstate ⟨*N*| and substituting the definition of |Ψ^(2)^(*t*, ω_*S*_)⟩ given in eq 3, we obtain the time-dependent coefficients of the state *N*
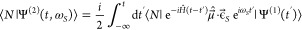
5The first order wave function can be expressed as well as an expansion on vibronic states |*J*⟩ weighted by the first order time-dependent coefficients, , which can be computed through the dynamics of the system wave function, initially in the GS, interacting with an incident radiation (practical details later). Equation 5 can be rewritten as
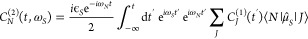
6where  is the monochromatic scattering field amplitude and  is the transition dipole moment operator along the direction of .

The second order wave function coefficients of a state *N* at the limit for *t* that tends to infinity is exactly the Inverse Fourier Transform (IFT) with respect to the scattered frequency of the first order coefficients, that is directly related to the Raman scattering intensity.^12^ From another perspective, the IFT of the first order coefficients as in eq 6 considering a finite time *t* collects the Raman scattering signal emitted until that moment, giving information on the ongoing Raman process. When the time dependence on the coefficients (i.e., without setting the limit to infinite) is kept, the Raman cross-section can be calculated as a function of time.

To get to this point the square modulus of the coefficients are computed as
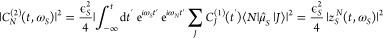
7enclosing in the term  the integral over *t*′. Numerically, the integral is calculated by a fast Fourier transform including in the integrand a Θ(*t* – *t*′) Heaviside function. The square modulus of the coefficients is the population of the molecular state *N* created by the scattered radiation with frequency ω_*S*_ from upper vibronic states. The time dependence of the population gives information on the total photons emitted until the observation time *t*. When a specific polarization direction for the incident field (η) is chosen and the scattered signal produced by the scattered field oriented along λ is selected, all the components  are calculated with indices λ, η that run over the Cartesian directions. When the  terms for all the combinations of λ and η are collected, they can be combined in the same way as the polarizability tensor to compute the Raman intensity with a precise polarization of the incident and scattered radiation, and an isotropic average of the molecular orientation is assumed. In this situation, eq 7 can be rewritten as
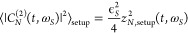
8indicating with  the population in the state *N* after isotropic orientational average for a specific illumination-detection setup, and with  the combination of  terms proper for that setup. For example, for incident radiation with linear polarization perpendicular to the detected scattered radiation and any polarization for the scattered radiation, the terms combine as
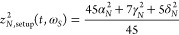
9with
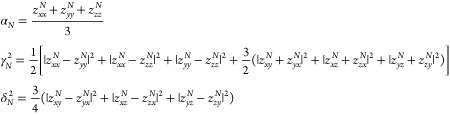
10

In order to compute the Raman cross-section as the ratio between the scattered energy and the incident radiation fluence we have to define these two quantities based on the theory developed so far. The whole population of the states involved in the scattering process is the sum of the square modulus of the second order coefficients over all the *N* vibronic states. The scattered energy is equal to the number of photons emitted at frequency ω_*S*_ multiplied by the emitted energy ω_*S*_ (in atomic units), where the number of scattered photons is equal to the product between the second order coefficients, representative of the states population achieved by a specific scattered field, and the number of scattered fields with frequency ω_*S*_ and amplitude ϵ_*S*_. It follows that the scattered energy over a small d*ω*_*S*_ frequency interval for a given illumination detection setup d*E*_*S*,setup_, can be written as a function of the second order coefficients as
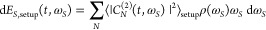
11where ρ(ω_*S*_) is the density of states of the scattered field with frequency ω_*S*_ and amplitude ϵ_*S*_. As mentioned, the number of emitted photons can be easily computed from eq 11 by dividing for the photon energy ω_*S*_. Substituting the square of the field amplitude with , the density of states of the scattered field with ,^11^ and the second order coefficients as in eq 8, we end up with the scattered energy per unit of frequency and per unit of solid angle
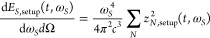
12On the other hand, the fluence of the incident field depends on its integral over time as
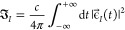
13Any field shape can be used in principle, even a known-by-points electric field. In this case we considered a Gaussian enveloped sinusoidal field with equation
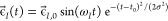
14centered in *t*_0_, with amplitude , width σ and frequency ω_*I*_. Integrating the incident electric field over time leads to the fluence of the incident field
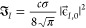
15

Dividing the scattered energy of eq 12 by the incident field fluence in eq 15, the cumulative time-dependent cross-section per unit of solid angle and per unit of scattered frequency is computed as a sum over the contribution given by each vibronic state *N*:
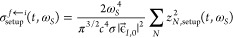
16With eq 16 we can not only recollect the overall Raman cross-section for a given illumination-detection setup, that can be also computed through strategies based on the transition polarizability tensor, but also the Raman signal as a function of time. In other words, this approach lets one know how progressively the Raman signal is accumulated and which is the time scale of the process.

### 2.2 Vibronic Wave Function

To obtain the Raman signal it is worthwhile to consider a proper wave function in the dynamics, dressing the electronic states with vibrational levels. The wave function, expanded on vibronic states, is considered within the BO approximation to separate electronic and nuclear motions. In this framework each vibronic eigenstate |*J*⟩ used in the previous section can be expressed as a product between an electronic (|ϕ_*el*_⟩) and a vibrational  wave function . Based on this approximation, the transition dipole moment between two vibronic states is expressed as
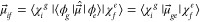
17with  and  being the initial and final vibrational levels which belong respectively to the ground (|ϕ_*g*_⟩) and the excited (|ϕ_*e*_⟩) electronic states. The vibrational levels are expanded within the normal mode approximation; e.g., anharmonic terms are neglected.^60,61^ Transition dipole moments between vibronic states that belong to the same electronic state are also neglected.

At this level the electronic and vibrational parts are decoupled so the Herzberg–Teller (HT) coupling has been included to recover for the transition dipole moment dependence on the normal mode coordinates, even if in an approximated way. Introducing the HT coupling allows inclusion of the dependence on nuclear coordinates in terms of a perturbative expansion of the electronic transition dipole due to the presence of a vibrational manifold. Therefore, the electronic transition dipole can be written as a Taylor expansion in the nuclear displacements around the nuclear equilibrium configuration ***Q***_0_(9)
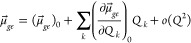
18where *k* runs over the normal mode coordinates. The first term of eq 18 is the electronic transition dipole moment calculated at the nuclear equilibrium position, while in the second term appears the transition dipole moment derivative with respect to the normal mode coordinates evaluated at the ground state equilibrium geometry. When eq 18 is included in eq 17, the new expression for the transition dipole moment between two generic vibronic states reads as
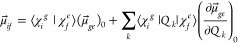
19The first term of eq 19 includes the Franck–Condon integral calculated between the final and initial vibronic states when the electric dipole moment is considered at the equilibrium normal mode coordinates. The second term accounts for the Herzberg–Teller contribution including a first order correction of the electric dipole moment with respect to the normal mode coordinates.^62^

### 2.3 Numerical Propagation

As mentioned above, within our approach the dynamics of the first order wave function is computed numerically. Practically the coefficients of the vibronic expansion are propagated through a second-order Euler algorithm as^55,63^
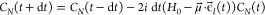
20in which d*t* is the time step chosen for the propagation, *H*_0_ is the time independent Hamiltonian that returns the energy of the state *N* while  accounts for the interaction with the incident electric field. The incident radiation can have in principle any profile since it is numerically defined. In this work we consider a Gaussian enveloped sinusoidal equation for the incident field as said before.

The greater part of the calculations has been performed considering the molecule as a closed quantum system. However, some others have been performed including the vibrational relaxation from the upper vibronic states to the lower level of the same electronic state. To this purpose the system wave function has been propagated through the Stochastic Schroedinger Equation (SSE)^43,45^ including nonradiative decay rate from the upper vibronic states toward the lower level of the electronic excited state. The SSE in a Markovian regime reads
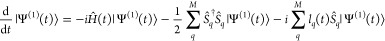
21where *q* runs over the number *M* of decay channels of the system and *l*_*q*_(*t*) is a white noise function associated with the Markov approximation needed to include the fluctuation induced by the environment. The first term in the r.h.s. of eq 21 represents the deterministic term related to the energy of the system and the interaction with the incident field, while the second and third terms are responsible for the decay processes guided by operators . According to previous works,^43,44,64,65^ relaxation has been included by the operator
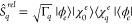
22which is responsible for the decay of the vibronic state  population on the state  where *q* is the vibrational level and *e* the electronic state. The decay rate Γ_*q*_ can be taken either as phenomenological parameters (as in the present case, see Computational Details) or estimated by ab initio simulations. Practically the coefficients dynamics are computed through the second-order Euler algorithm as in eq 20 while the stochastic terms of SSE are included by means of a quantum jumps algorithm.^43^ This algorithm is based on discontinuous trajectories. At each time step during the dynamics, the probability of the jump to occur associated with the decay operator  and given by  is estimated. Whether the jump actually occurs or not is decided by a Monte Carlo algorithm. When the jump happens, the wave function |Ψ^(1)^(*t*)⟩ is replaced by  and normalized; on the contrary it is only normalized. The density matrix evolution is obtained by averaging over several of these trajectories. For further details see ref (43).

The coefficients dynamics of each trajectory has been employed to compute the second order coefficients. Varying the polarization of the incident field and the scattered radiation, we computed all the terms  needed to calculate, for a given illumination-detection setup and for each trajectory, the time-dependent Raman cross-section  and finally the average on all the realizations has been taken as
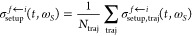
23The Raman cross-section is a measurement of the amount of population generated in each final vibronic state by scattered fields with different frequencies ω_*S*_. The population computed from a single trajectory is meaningless in itself but when the average over a number of realizations is taken, the SSE converges to the Lindbland master equation results.^43,66^

### 2.4 Computational Details

The approach presented in the previous sections has been applied to porphyrin, as a test case. The quantum chemical calculations have been performed through DFT methods at the B3LYP/6-31G level of theory using Gaussian 16.^67^ The ground state and first excited state were optimized, and then the vibrational calculation was performed on both potential energy surfaces. To obtain a proper wave function for the system, the two electronic states have been dressed with 109 vibrational states each (the ground vibronic state and the first vibronic level for each normal mode). We computed the FC and HT integrals of the molecule between all the vibronic states belonging to different electronic states through FCclasses code.^62,68^ The vibronic analysis has been carried out within the adiabatic Hessian model which takes into account the Duschinsky mixing and the frequency changes between ground and excited state. The FC and HT integrals have been employed to compute the transition dipole moments as in eq 19. The system wave function is approximated by including only one excited electronic state, thus when in nonresonance conditions there could be significant differences on the spectra shape and intensity compared with the experimental results. However, the focus of this work is on the time scale of the process and on the interplay with fluorescence.

The dynamics of the first order system wave function has been computed through the WaveT code.^43,69^ During the propagation the system interacts with an incident electric field shaped as a Gaussian enveloped sinusoidal signal, as mentioned above. Three different simulations for each field setup have been computed varying the incident field direction along *x*, *y*, and *z* axes in order to have all the information needed for the calculation of the term  as in eq 9.

The dynamics length is 24 ps in almost all the calculations and the time step considered is 0.024 fs. When it is not differently specified, the field has a Gaussian enveloped sinusoidal shape (see sec. 2.3) with σ = 256 fs, ϵ_*I*,0_ = 5.14 × 10^5^ V/m, centered at *t*_0_ = 968 fs. In nonresonant conditions, the excitation energy is ω_*I*_ = 2.04 eV, which is 0.24 eV less than the vertical transition frequency *S*_1_ ← *S*_0_. After the calculation of the coefficients dynamics, the cumulative Raman cross-section was computed at different times, through eq 16. The convergence of the spectrum can be checked as explained in ref (21).

In the simulation with the vibrational relaxation, 50 repetitions of the dynamics were computed with vibrational relaxation equal to 1.8 ps.^70^ Convergence with respect to the number of employed trajectories when including vibrational relaxation in resonance conditions has been confirmed comparing the Raman spectra at 2.4 ps computed with either 50 or 100 trajectories. The result is reported in Figure S1 of the Supporting Information.

## 3 Results and Discussion

**Figure 1:**
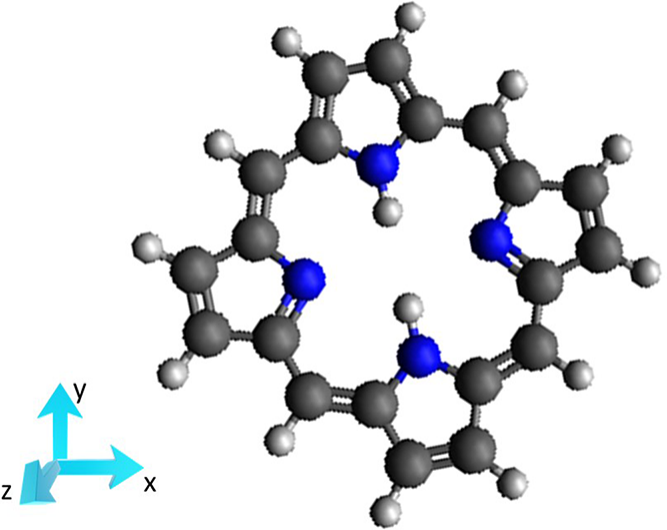
Structure of porphyrin molecule and orientation employed for the calculations.

The procedure developed to compute Raman scattering cross-section has been applied to porphyrin, laying in the *xy* plane as Figure 1 shows. The calculation of the cumulative time-dependent Raman cross-section has been performed by employing two different field frequencies to study the nonresonance and resonance conditions with respect to the electronic excitation energy. Moreover also the field duration and the presence of the vibrational relaxation have been exploited.

To validate the numerical strategy, we compared porphyrin Raman spectrum computed through our TD method in nonresonant conditions with the spectrum computed with a time independent strategy, by employing the FCclasses code.^21,68^ The results, reported in Figure S2, show a good agreement, therefore confirming the correctness of our strategy.

Moreover a comparison with the experimental Raman spectrum of porphyrin^71^ is reported in Figure S3. Apart from an overall rescaling of the frequencies, which is expected for harmonic spectra,^72,73^ the agreement is good. The remaining discrepancies (beside the frequency rescaling), may be due to the different environments (the experiment is conducted in CH_2_Cl_2_, while the calculation is performed in vacuum), to anharmonic effects beside the rescaling and to inherent limitation of the DFT xc functional employed.

### 3.1 Nonresonance Conditions

In all the calculations reported in this section, the first order coefficients of the system wave function have been computed when the system is initially in the ground electronic state and then interacts with an incident electric field nonresonant with the electronic transition as explained before. After the computation of the coefficients dynamics varying the incident field direction along *x*, *y*, and *z* axes, three different scattering directions (along *x*, *y*, and *z*) have been considered for each incident field direction to compute the time-dependent Raman cross-section in atomic units as in eq 16. Since the electronic transition dipole moment *S*_1_ ← *S*_0_ has the major contribution along the *x* and *y* axes, the terms with incident and/or scattered field along the *x* and *y* axes give the largest contribution to the Raman cross-section.

#### Neglecting Vibrational Relaxation

**Figure 2:**
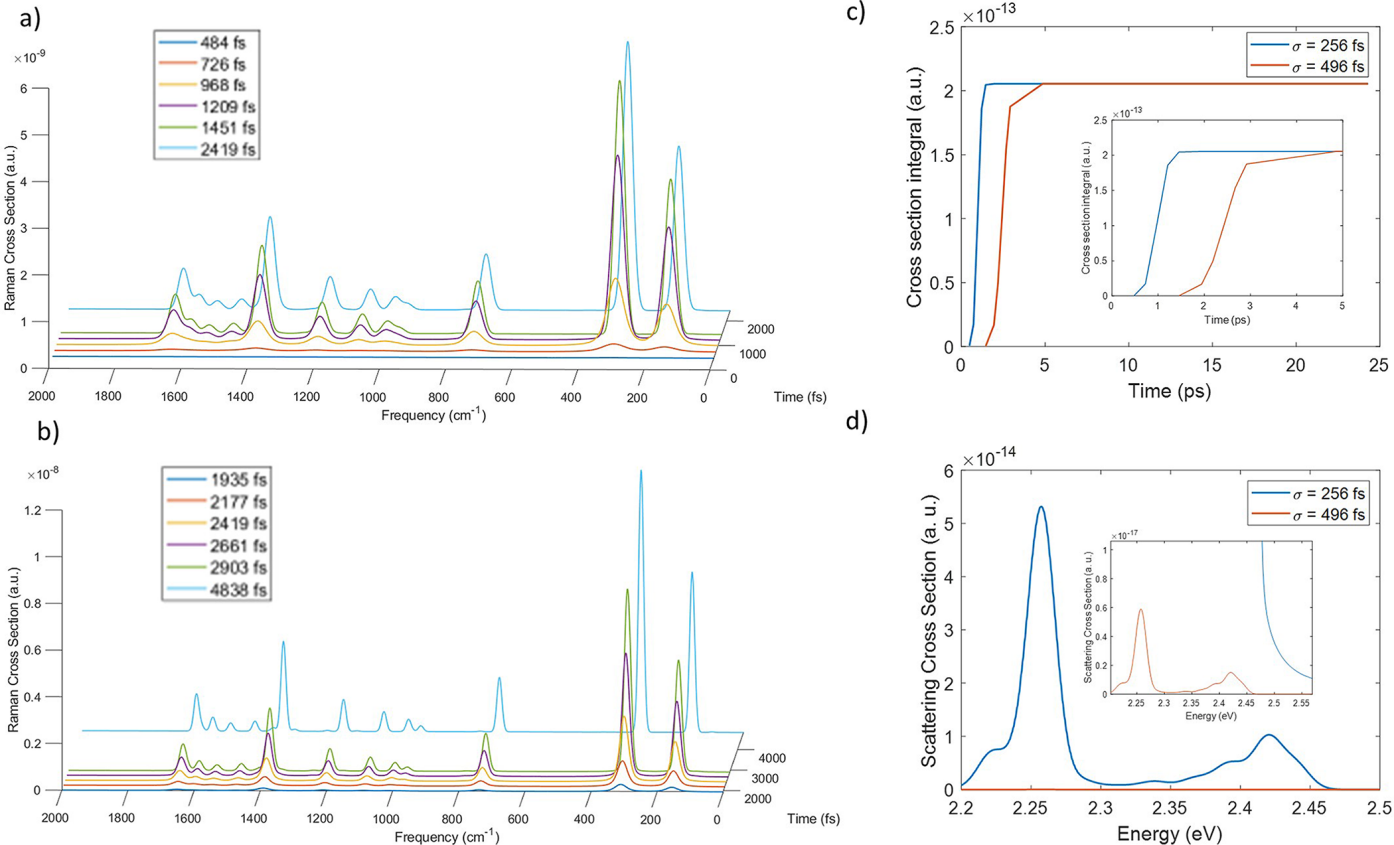
Time dependent Raman cross-section in atomic units at six different time delays with respect to the beginning of dynamics, with incident pulse in nonresonant conditions with σ = 256 fs (a) and with σ = 496 fs (b). (c)Time dependent Raman cross-section integral of the peak at 738 cm^–1^ in the case of field with σ = 256 fs and with σ = 496 fs. (d) Scattering signal at the end of dynamics in the resonance region when the system interacts with a nonresonant field in which σ is 256 fs or 496 fs. The inset is a magnification of the emitted signal generated with an incident field that has σ = 496 fs.

The Raman spectrum as a function of time has been reported in Figure 2a for six different time delays with respect to the beginning of dynamics.

The results show how the Raman intensity is built during and after the interaction with the incident field. Before 484 fs, far from the maximum intensity of the electric field, the Raman intensity is negligible, but before reaching the center of the field at 968 fs, the Raman signal has been already generated due to the short time taken by the process. After 1451 fs from the beginning of the dynamics quite all the Raman intensity is recovered, even if the pulse has not switched off completely. The last spectrum here reported, after 2.4 ps from the beginning of the dynamics, when the pulse is approximately null, is totally equal to the final spectrum computed at 24 ps: all the Raman contribution to emission is achieved in the first hundreds femtoseconds from the interaction with the incident field.

These observations are confirmed by the second column of Table 1 which reports the integral of the peak at 738 cm^–1^ as a function of time, representing the amount of Raman signal collected until the observation time from the beginning of the dynamics without the dependence on the frequency spread signal. The integrals highlight the fast increasing intensity in the first few hundreds of femtoseconds around the maximum field intensity followed by a constant value, a signal that the maximum emitted intensity in this region has been achieved. One possible way to interpret the results is that Raman scattering persists as long as the wave function coefficients have memory of the pulse frequency or, from a more physical viewpoint, until the vibronic excited states are transiently perturbed by the presence of the incident field. After the end of the interaction with the field no more Raman signal is collected since the process, very quickly, is exhausted.

**Table 1:** Cross-Section Integrals of the Peak at 738 cm^–1^ as a Function of Time in Three Cases: with σ = 256 fs (from Figure 2a), with σ = 496 fs (from Figure 2b), and with σ = 256 fs Including also Vibrational Relaxation (from Figure 3)

Time	Cross-section integral (σ = 255 fs)	Time	Cross-section integral (σ = 496 fs)	Time	Cross-section integral (vib. relaxation)
fs	a.u.	fs	a.u.	fs	a.u.
484	6.7250 × 10^–16^	1457	4.3633 × 10^–16^	484	6.7236 × 10^–16^
726	1.7272 × 10^–14^	1943	1.6738 × 10^–14^	726	1.7277 × 10^–14^
968	9.9813 × 10^–14^	2186	4.9304 × 10^–14^	968	9.9807 × 10^–14^
1209	1.8585 × 10^–13^	2429	1.0129 × 10^–13^	1209	1.8582 × 10^–13^
1451	2.0457 × 10^–13^	2671	1.5386 × 10^–13^	1451	2.0456 × 10^–13^
1936	2.0538 × 10^–13^	2915	1.8756 × 10^–13^	1936	2.0536 × 10^–13^
2428	2.0538 × 10^–13^	4857	2.0538 × 10^–13^	2428	2.0536 × 10^–13^
24289	2.0538 × 10^–13^	24289	2.0538 × 10^–13^	4856	2.0536 × 10^–13^

#### Increasing Field Time Duration

Another calculation has been performed increasing the field time duration by modifying the width of the pulse with σ = 496 fs and moving the center of the pulse at 2419 fs. In this case the pulse is larger so it is even closer to a monochromatic pulse than the previous one. The Raman spectrum at different time delays from the beginning of the dynamics is reported in Figure 2b. The vibronic states that participate in the Raman spectrum are the same as in the previous case, while the time scale of the signal generation is longer and comparable to the width of the incident pulse employed here. The signal peaks are narrower and well separated due to the incident field shape, closer to a monochromatic radiation than in the previous one. As a consequence, these results highlight the dependence of the spectrum line shape on the electric field shape and, in particular in this case, on the duration of the pulse. At last the intensity of the Raman signal in time can be compared with the previous one by removing the frequency dependence through the integration of the peak at 738 cm^–1^ as reported in Figure 2c and in the fourth column of Table 1. These results show that the amount of scattered radiation is quite the same at the end of the dynamics although the time of the process is longer in the case of a longer incident pulse. On the other hand in the fluorescence emission region (around the transition *S*_1_ ← *S*_0_ energy) the emitted signal has a lower intensity when a longer lasting field is employed, as represented in Figure 2d, because the smaller frequency amplitude of the electric field can excite (nontransiently, as needed for fluorescence emission) less amount of the excited states population.

#### Including Vibrational Relaxation

**Figure 3:**
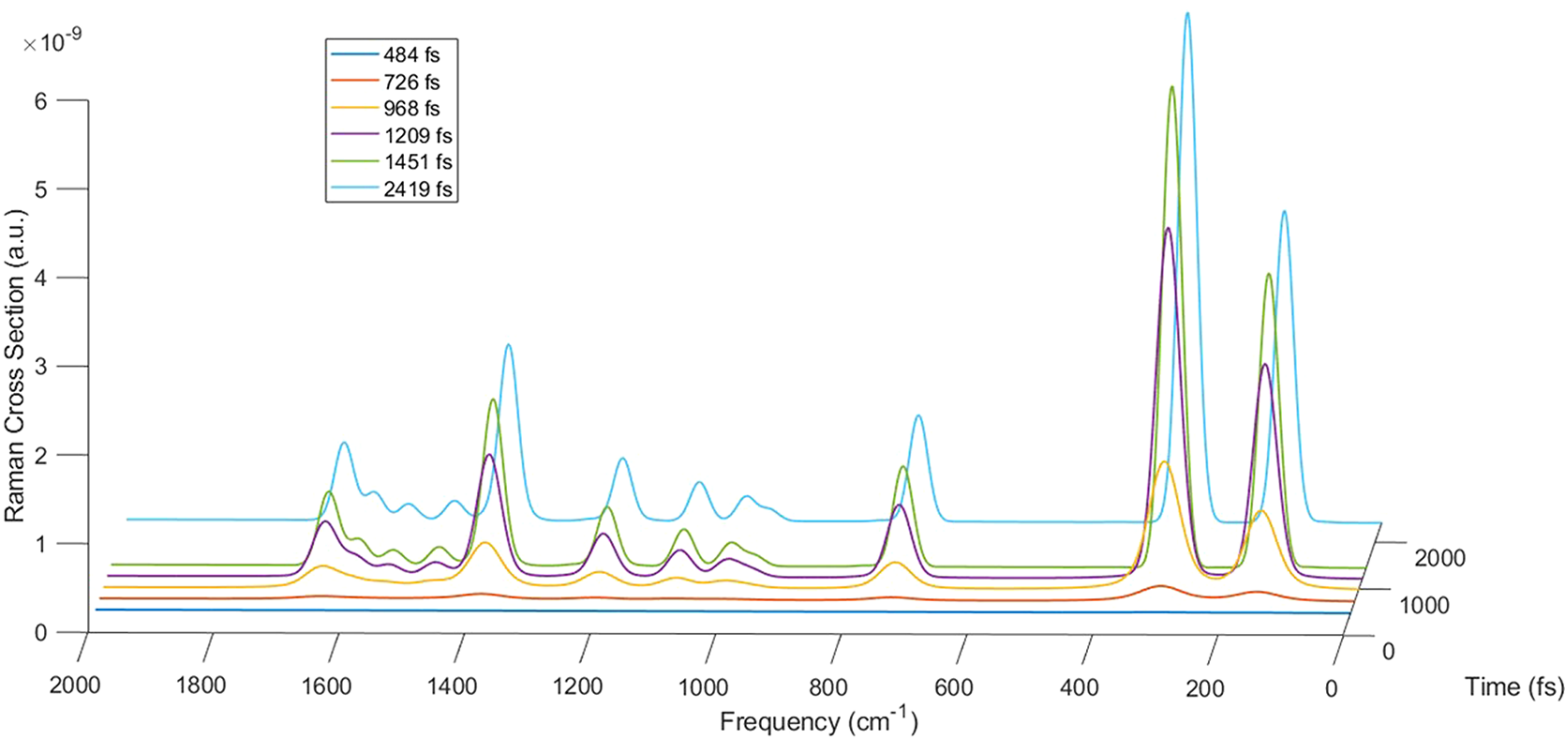
Time-dependent Raman cross-section in atomic units at six different time delays with respect to the beginning of dynamics, with incident pulse in nonresonant conditions, σ = 256 fs and including vibrational relaxation.

Another calculation of porphyrin Raman cross-section has been carried out including vibrational relaxation. To this point, field features have been kept as in the first calculation reported in this section (nonresonant electric field with σ = 256 fs), and the vibrational relaxation has been included from upper vibronic states to the lowest level of the correspondent electronic state with the phenomenological lifetime of 1.8 ps.^70^ 50 trajectories have been computed through SSE equation using in each case three incident field directions (along *x*, *y*, *z* axes) and the dynamics of each simulation is 4.8 ps long. For each trajectory the time-dependent Raman cross-section has been computed, and then they have been averaged to get the mean Raman cross-section. The time evolution of the Raman cross-section including vibrational relaxation, reported in Figure 3, looks very close to the results in which it is neglected (Figure 2a) as expected since the time scale of the process seems faster than the vibrational decay. This result is confirmed by the time-dependent integral of the peak at 738 cm^–1^, the values of which, reported in column six of Table 1, totally resemble those of the deterministic dynamics (without vibrational relaxation) reported in the second column of the same table. As a conclusion, the introduction of vibrational relaxation is not needed, at least in nonresonant conditions, due to the different time scale of the processes.

### 3.2 Resonance Conditions

The results reported in this section are related to calculations carried out with the incident field frequency that matches the porphyrin vertical excitation energy (ω_*I*_ = 2.28 eV), while the maximum amplitude is equal to 5.14 × 10^5^ V/m, the pulse width has been varied as in the calculations of section 3.1. The time-dependent scattering cross-section has been computed on the basis of the system dynamics when the incident field is polarized alternately along *x*, *y*, and *z* axes. For these calculations in resonant conditions, we refer to the results as to *scattering* cross-sections, rather than to Raman cross-sections given that the Raman scattering and the fluorescence emission lay in the same spectral range. Thus, in principle the two contributions may be mixed and the scattering cross-section is no more the result of the pure Raman contribution.

#### Neglecting vibrational relaxation

**Figure 4:**
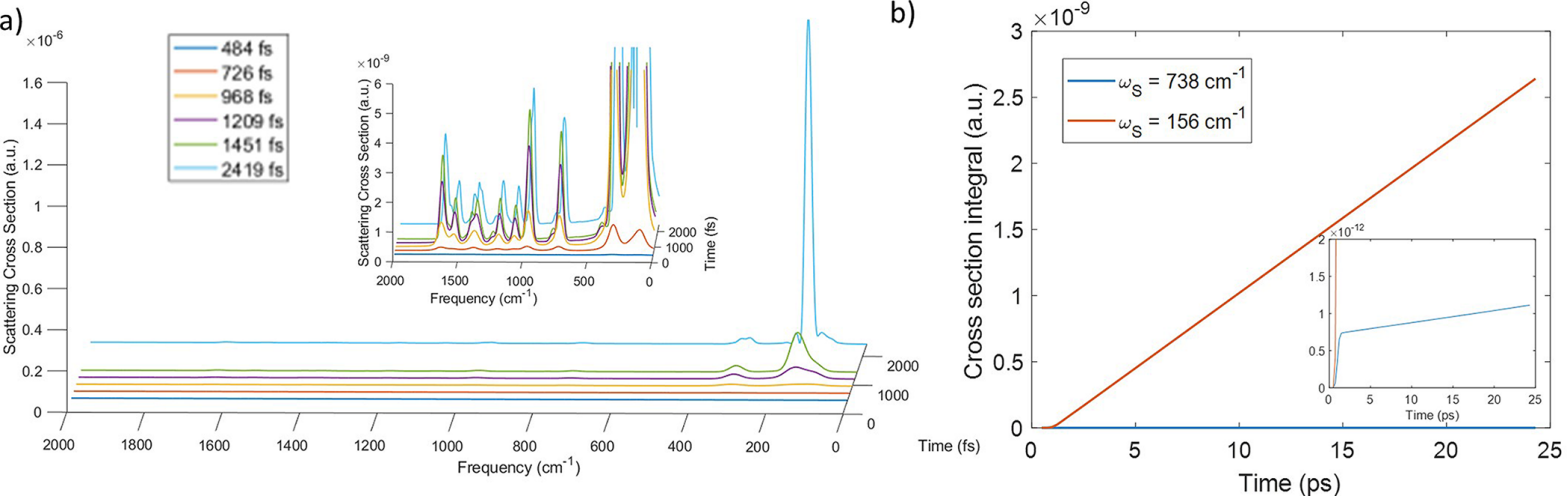
(a) Time-dependent scattering cross-section in atomic units at six different time delays with respect to the beginning of dynamics, with incident pulse in resonance conditions and σ = 256 fs. The inset shows a magnification of the part of the spectrum with lower intensity. (b) Time-dependent scattering cross-section integral of the peaks at 738 cm^–1^ and the peak at 156 cm^–1^ of the spectra in panel a.

In the first calculation the width of the pulse is σ = 256 fs and it is centered at *t*_0_ = 968 fs, as in the first calculation in nonresonance conditions. In Figure 4a the time-dependent scattering cross-section accumulated at six different times from the beginning of dynamics has been reported. Differently than in nonresonance conditions, in this case the scattering cross-section does not achieve its maximum after 1451 fs but at 2419 fs the intensity is further increasing, as an effect of the fluorescence emission. Moreover the vertical transition matches a few vibronic states in the excited state that give the largest contribution to the scattering spectrum. Also the speed at which the peaks’ intensity increases is different, and it is larger for the peak at 156 cm^–1^, in which the fluorescence emission prevails over the Raman scattering signal, as also Figure 4b shows in terms of the integral of the scattered signals at 156 cm^–1^ and 738 cm^–1^. This figure highlights both the rate of signal emission and the order of magnitude of the intensity achieved within this time scale, which are very different.

#### Increasing Field Time Duration

**Figure 5:**
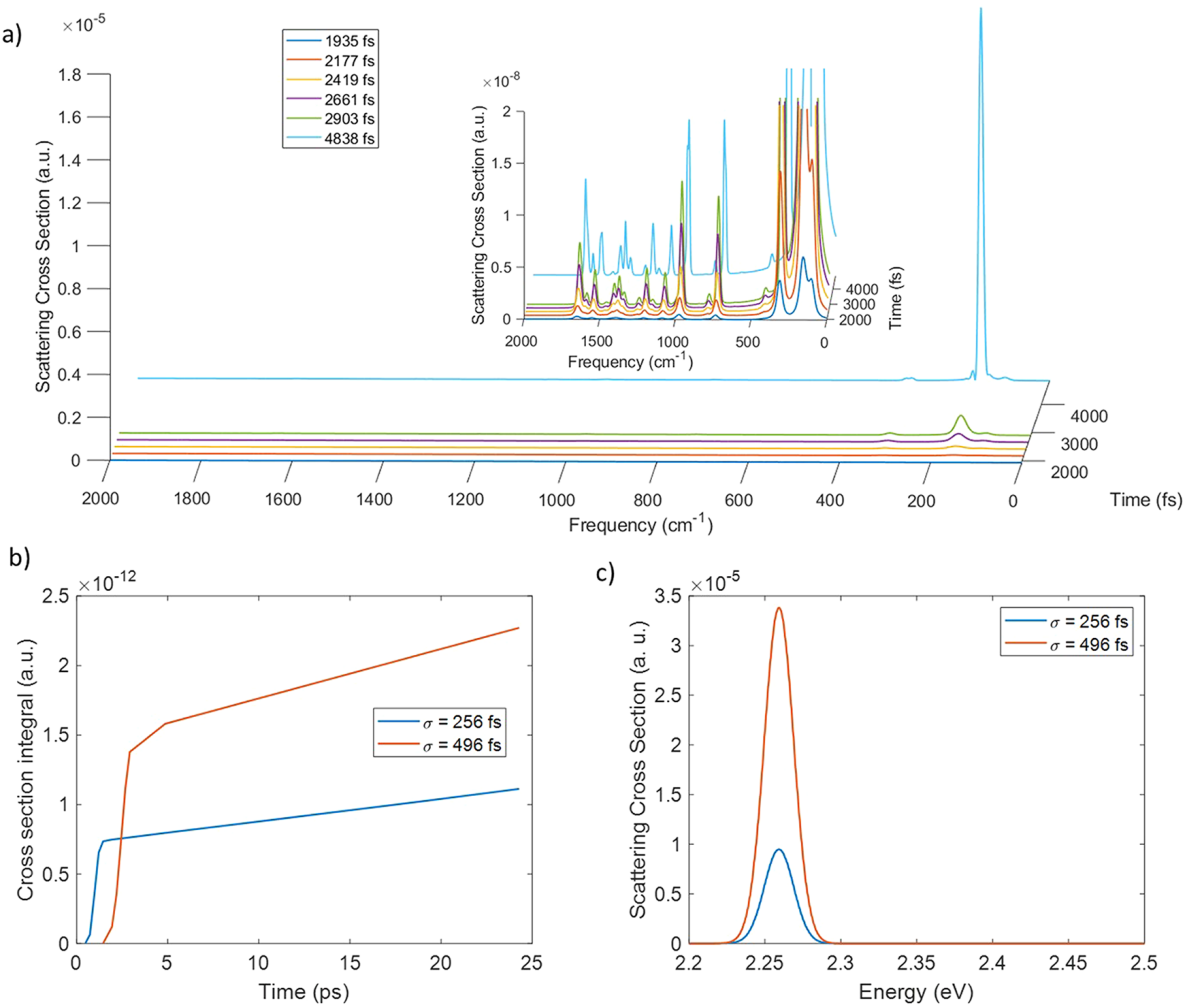
(a) Time-dependent scattering cross-section in atomic units at six different time delays with respect to the beginning of dynamics, with incident pulse in resonance conditions and σ = 496 fs. The inset shows a magnification of the part of the spectrum with lower intensity. (b) Time-dependent scattering cross-section integral of the peak at 738 cm^–1^ in the case of field with σ = 256 fs and with σ = 496 fs. (c) Scattering signal at the end of dynamics in the resonance region when the system interacts with a resonant field which σ is 256 fs or 496 fs.

As it has been done in nonresonant conditions, we tested the influence of a longer pulse on the time-dependent scattering cross-section in resonance conditions, by using a larger width of the pulse (σ = 496 fs). The cumulative scattering cross-section for six different times, reported in Figure 5a, shows thinner peaks than the previous simulation and a longer time scale as expected. Moreover the peak at 156 cm^–1^ is enhanced also in this case. The comparison of these results with those obtained with a shorter pulse in terms of the integral of the peak at 738 cm^–1^ are shown in Figure 5b. In this case, differently than in the nonresonant case, the duration of the pulse has an effect on the total scattered signal since the cross-section increases with the longer pulse. This is due to the sum of the two processes that are going on: on one hand the pure Raman scattering, that should remain constant with the pulse duration as in nonresonant conditions, and the fluorescence intensity that strongly depends on the amount of population created on the excited state.

In Figure 5c the convoluted scattering cross-section at 24 ps has been reported for the two simulations with different field widths. In this case the trend as a function of the field duration is opposite with respect to the one in nonresonance conditions since, as mentioned before, the longer is the resonant field the more intense is the scattered signal in the emission region in which both the Raman and fluorescence contributions are mixed. Moreover, on the basis of the results, here the fluorescence contribution prevails.

Practically, in resonant conditions, a relevant population on vibronic states that belong to the excited electronic state, is generated, and it persists even after the incident field is switched off giving a non-negligible contribution to the fluorescence emission. Due to the longer time scale of the process, the fluorescence emission at 24 ps is not exhausted as the Raman scattering which is a faster process as it does not need to create a population on the excited state to occur, but it is the result of the quantum superposition of vibronic states that belong to two different electronic states. By computing a longer dynamics for the first order coefficients, all the emission intensity can be retrieved, but that is beyond the aim of this work.

#### Including vibrational relaxation

**Figure 6:**
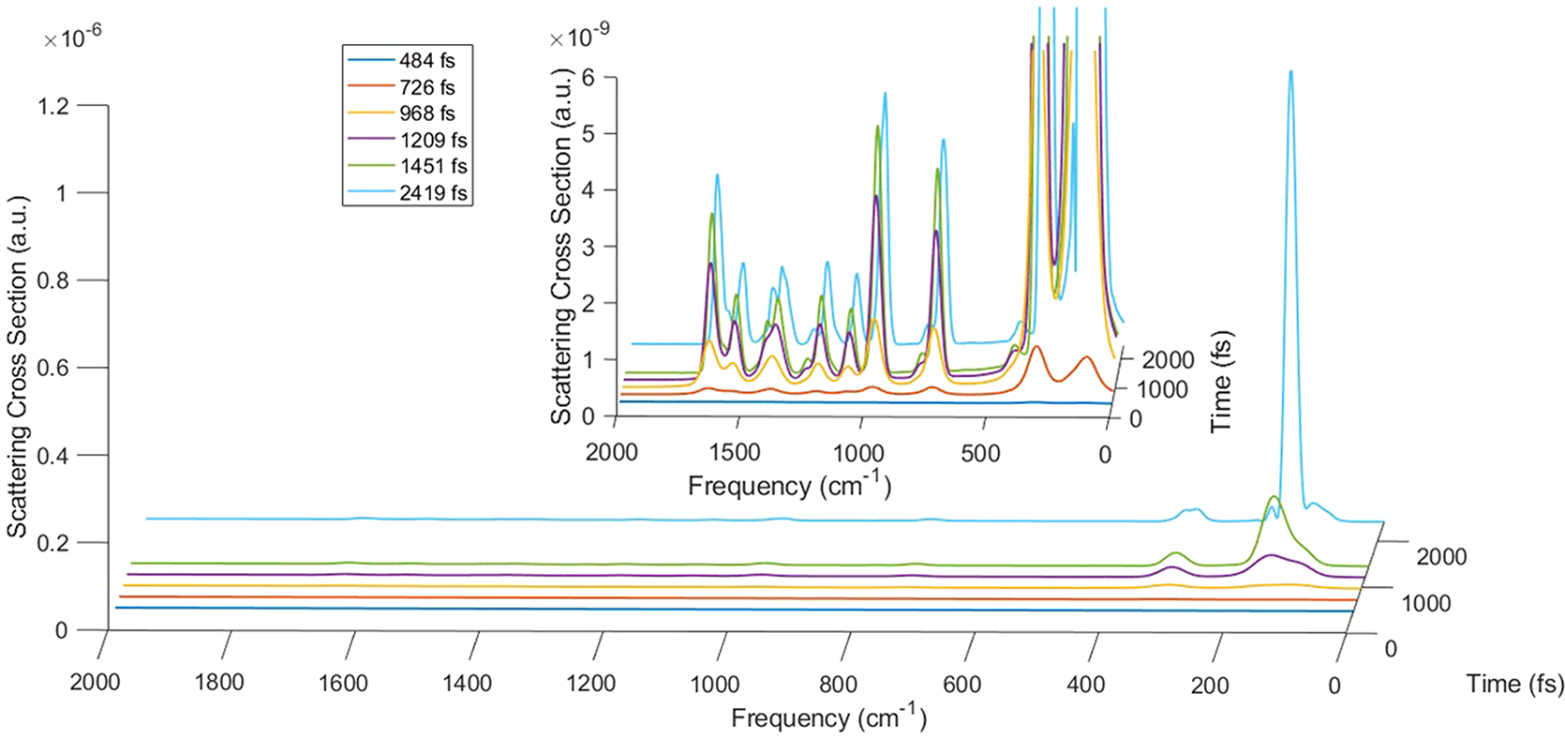
Time-dependent scattering cross-section in atomic units at six different time delays with respect to the beginning of dynamics, with incident pulse in resonant conditions, σ = 256 fs and including vibrational relaxation.

**Figure 7:**
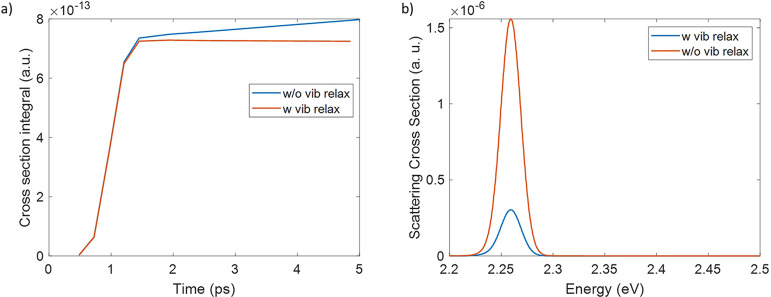
(a) Time-dependent cross-section integral of the peak at 738 cm^–1^, with incident pulse in resonant conditions, σ = 256 fs, including or without including vibrational relaxation. (b) Scattering signal at 4.8 ps when the system interacts with a resonant field including or without including vibrational relaxation.

Finally, in resonant conditions, a simulation has been carried out including the vibrational relaxation from upper vibronic states to the lower level of the corresponding electronic state. To this point 50 trajectories have been computed for a dynamics 4.8 ps long, considering three directions of the electric field along *x*, *y*, and *z* axes. After computing the time-dependent cross-section for each trajectory, the average of them has been taken. The resulted scattering cross-section, reported in Figure 6, shows a profile close to the one in absence of vibrational relaxation. However, the maximum intensity is lower. This can be better appreciated by the integral of the peak at 738 cm^–1^ (Figure 7a) which shows a flat trend after the maximum emission has been achieved while without including relaxation the emission is still increasing. Moreover the convoluted emission spectrum at 4.8 ps shows a lower intensity when the vibrational relaxation is included, as Figure 7b shows, since the population of the vibronic state with frequency equal to 156 cm^–1^ (which gives the largest contribution to the scattering spectrum) has decayed.

## 4 Conclusion

A computational procedure to calculate Raman scattering cross-section as a function of time has been presented, based on the first order coefficients of the wave function computed after the interaction with an incident electric field that can have any possible shape. The main advantage of this approach is to give a time-dependent picture of the Raman process, not only because it is based on the time-dependent strategies developed so far, but especially for the possibility to compute the cumulative Raman signal at different times of the process. Giving a time-dependent picture of the process allows explicit inclusion of phenomena that have the same time scale of the Raman scattering, such as the interaction with an incident pulse with femtoseconds to picoseconds duration and any possible profile, and the vibrational relaxation, which is particularly relevant in resonant conditions to simultaneously simulate Raman and fluorescence emission. Moreover, in a future perspective, this method allows inclusion of the mutual interaction with a nearby plasmonic nanoparticle^54^ that not only affects the intensity of the Raman signal but may change also the shape and the time scale of the process due to the local field felt by the molecule. Based on this premise, our approach will be useful also to simulate results of SERS experiments in a time-dependent fashion.

This procedure has been applied to porphyrin to calculate the time-dependent Raman cross-section in resonant and nonresonant conditions exploiting also the effect of the electric field duration and the presence of vibrational relaxation. In nonresonant conditions the Raman scattering is perfectly distinguishable from the fluorescence emission as they appear in different spectral regions, and so as well the vibrational relaxation does not affect the Raman signal since it occurs on a longer time scale. On the side of the pulse shape, the field duration does not alter the total Raman signal gathered at the end of the process, while the fluorescence region is strongly affected by it.

On the other hand, when in resonant conditions the Raman scattering is rapidly followed by the fluorescence emission which is a more intense phenomenon that occupies the same spectral region, so the distinction between the two contributions is not trivial. Also the spectrum shape is very different than the one in nonresonant conditions due to the great enhancement of the peaks generated from the excited vibronic states around the vertical excitation energy. In these conditions, the presence of the vibrational relaxation is needed since it occurs in the time scale of the fluorescence emission, and indeed the spectrum is strongly influenced by the decay process. Our approach gives information on the interplay between Raman scattering and fluorescence emission in terms of relative weight of the two processes on the generation of the spectrum line shape even if the two contributions are not quantitatively distinguishable.

## Supporting Information Available

Convergence of Raman spectrum with 50 trajectories, comparison between porphyrin Raman spectrum computed with the present TD method and with a time-independent strategy, comparison between porphyrin Raman spectrum computed with the present TD approach and the experimental one (PDF)
