# Supplementary material for: Time Resolved Raman Scattering of Molecules: A Quantum Mechanics Approach with Stochastic Schroedinger Equation
Source: J Phys Chem A. 2022 Oct 24;126(43):8088–100. doi: 10.1021/acs.jpca.2c05245 (PMC9639147; doi:10.1021/acs.jpca.2c05245)
Supplement: Supplementary file 1 — jp2c05245_si_001.pdf [file jp2c05245_si_001.pdf]

Supporting Information for Time Resolved Raman Scattering of Molecules: a Quantum Mechanics Approach with Stochastic Schroedinger Equation

Giulia Dall'Osto† and Stefano Corni*,†,‡

†*Department of Chemical Sciences, University of Padova, via Marzolo 1, Padova, Italy, 35100*

‡*CNR Institute of Nanoscience, via Campi 213/A, Modena, Italy, 41100*

E-mail: stefano.corni@unipd.it

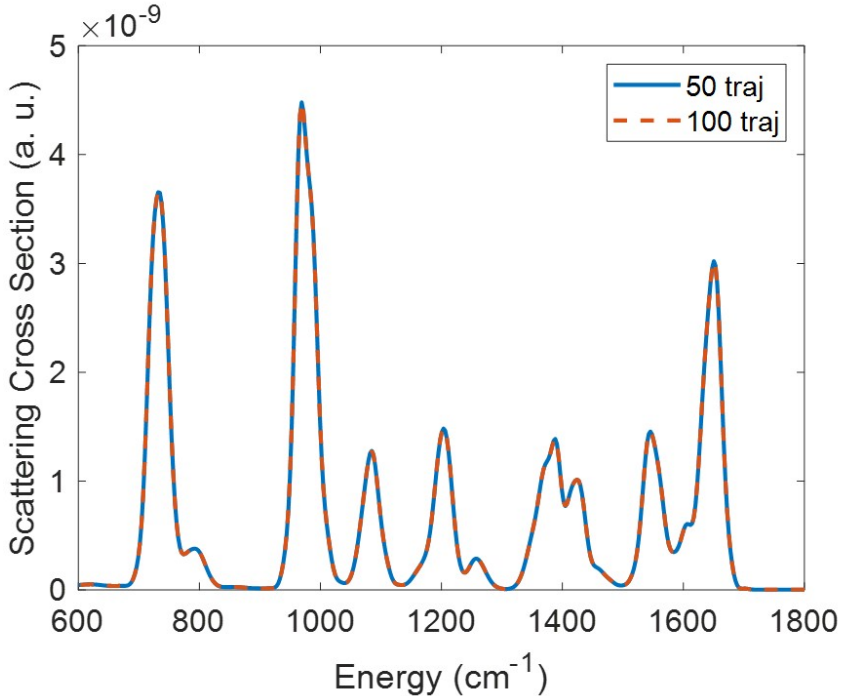

Figure S1: Comparison between Porphyrin Raman spectrum computed through our TD approach at 2.4 ps using 50 trajectories (blu, straight line) and using 100 trajectories (orange, dashed line). The calculations have been carried out in resonance conditions,with the incident field matching the vertical transition and including vibrational relaxation through SSE.

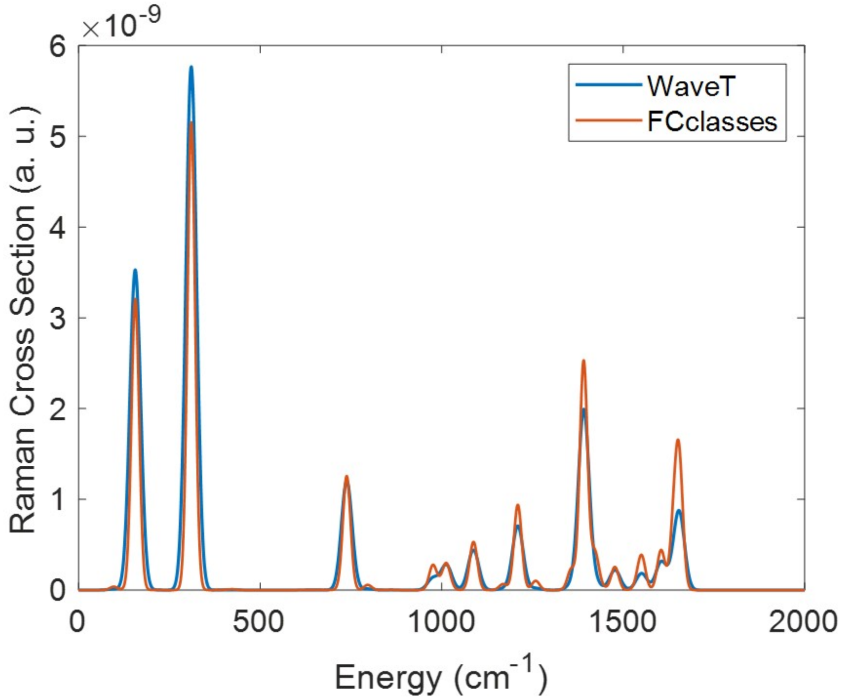

Figure S2: Comparison between Porphyrin Raman spectrum computed through our TD approach (at 24 ps) and with a time independent strategy (through FCclasses code).[1,2] Both spectra are reported with the incident radiation in non-resonance conditions, with energy 0.24 eV lower than the vertical transition.

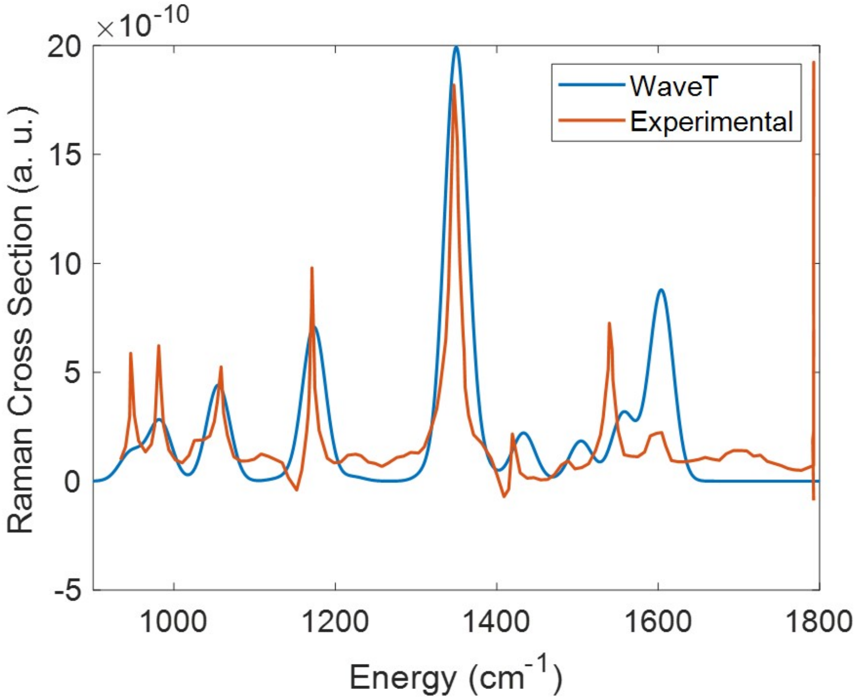

Figure S3: Comparison between Porphyrin Raman spectrum computed through our TD approach (at 24 ps) and the experimental Resonance Raman spectrum obtained in CH_2Cl_2 solution.[3] The theoretical spectrum has been re-scaled by a factor 0.97[4] (scaling factor for B3LYP exchange-correlation functional) to correct the vibrational frequencies computed at DFT level. The theoretical calculation has been performed in non-resonance conditions with the incident radiation energy 0.24 eV lower than the vertical transition.

References

(1) Santoro, F.; Cerezo, J. FCclasses3, a code for vibronic calculations. `http://www.iccom.cnr.it/en/fcclasses`, 2022; [Online; accessed 27-July-2022].

(2) Santoro, F.; Cappelli, C.; Barone, V. Effective Time-Independent Calculations of Vibrational Resonance Raman Spectra of Isolated and Solvated Molecules Including Duschinsky and Herzberg–Teller Effects. *J. Chem. Theory Comput.* **2011**, *7*, 1824–1839.

(3) Taniguchi, M.; Mass, O.; Boyle, P. D.; Tang, Q.; Diers, J. R.; Bocian, D. F.; Holten, D.; Lindsey, J. S. Structural studies of sparsely substituted synthetic chlorins and phorbines establish benchmarks for changes in the ligand core and framework of chlorophyll macrocycles. *J. Mol. Struct.* **2010**, *979*, 27–45.

(4) Irikura, K. K.; Johnson, R. D.; Kacker, R. N. Uncertainties in scaling factors for ab initio vibrational frequencies. *J. Phys. Chem. A* **2005**, *109*, 8430–8437.
